# Year-round spatiotemporal distribution pattern of a threatened sea duck species breeding on Kolguev Island, south-eastern Barents Sea

**DOI:** 10.1186/s12898-020-00299-2

**Published:** 2020-05-25

**Authors:** Thiemo Karwinkel, Ingrid L. Pollet, Sandra Vardeh, Helmut Kruckenberg, Petr Glazov, Julia Loshchagina, Alexander Kondratyev, Benjamin Merkel, Jochen Bellebaum, Petra Quillfeldt

**Affiliations:** 1 Department of Animal Ecology and Systematics, Justus Liebig University Giessen, Heinrich-Buff-Ring 26-32, 35392 Giessen, Germany; 2 Institute of Avian Research “Vogelwarte Helgoland”, An der Vogelwarte 21, 26386 Wilhelmshaven, Germany; 3 Institute of Biology and Environmental Sciences, Carl von Ossietzky University Oldenburg, Carl-von-Ossietzky-Straße 9-11, 26129 Oldenburg, Germany; 4 Institute for Waterbird and Wetlands Research (IWWR) e.V. Germany, Am Steigbügel 3, 27283 Verden (Aller), Germany; 5 Institute of Geography RAS, Staromonetniy per 29, 119017 Moscow, Russia; 6 Institute of Biological Problems of the North FEB RAS, Portovaya str. 18, 685000 Magadan, Russia; 7 Fram Centre, Norwegian Polar Institute, P.O. Box 6606, Langnes, 9296 Tromsø, Norway

**Keywords:** Long-tailed duck, *Clangula hyemalis*, Baltic Sea, Geolocation, Sea duck, Conservation, Russian Arctic

## Abstract

**Background:**

The long-tailed duck (*Clangula hyemalis*) was categorized as ´Vulnerable` by the IUCN after a study revealed a rapid wintering population decline of 65% between 1992–1993 and 2007–2009 in the Baltic Sea. As knowledge about the European long-tailed duck’s life cycle and movement ecology is limited, we investigate its year-round spatiotemporal distribution patterns. Specifically, we aimed to identify the wintering grounds, timing of migration and staging of this population via light-level geolocation.

**Results:**

Of the 48 female long-tailed ducks tagged on Kolguev Island (western Russian Arctic), 19 were recaptured to obtain data. After breeding and moulting at freshwater lakes, ducks went out to sea around Kolguev Island and to marine waters ranging from the White Sea to Novaya Zemlya Archipelago for 33 ± 10 days. After a rapid autumn migration, 18 of 19 birds spent their winter in the Baltic Sea and one bird in the White Sea, where they stayed for 212 ± 3 days. There, they used areas known to host long-tailed ducks, but areas differed among individuals. After a rapid spring migration in mid-May, the birds spent 23 ± 3 days at sea in coastal areas between the White Sea and Kolguev Island, before returning to their freshwater breeding habitats in June.

**Conclusions:**

The Baltic Sea represents the most important wintering area for female long-tailed ducks from Kolguev Island. Important spring and autumn staging areas include the Barents Sea and the White Sea. Climate change will render these habitats more exposed to human impacts in the form of fisheries, marine traffic and oil exploitation in near future. Threats that now operate in the wintering areas may thus spread to the higher latitude staging areas and further increase the pressure on long-tailed ducks.

## Background

Sea ducks are waterfowl living in marine environments outside of the breeding season, mostly in shallow coastal waters or offshore banks [1, 2]. The majority of sea duck populations breed in the Arctic, where they nest on the ground close to small freshwater lakes [1, 3]. Due to the inaccessibility and the vast size of their breeding range, knowledge about their population dynamics from the breeding grounds is limited [4–6]. One of the world´s most important wintering sites for sea ducks is the Baltic Sea [2]. Offshore surveys have shown that sea ducks wintering in the Baltic Sea experienced population declines of 60% between 1992–1993 and 2007–2009 [7]. The sea duck decline is not limited to European populations. A decline of 50% was also observed for the five most common sea duck species in North America between the mid-1970s and 1996 [8].

The long-tailed duck (*Clangula hyemalis*) is the most abundant sea duck species [9–12]. Its wintering population in the Baltic Sea declined by 65% from around 4.3 to 1.5 million birds between 1992–1993 and 2007–2009, following the general pattern of other sea ducks [7]. These observations are particularly alarming, as it is assumed that 90% of the long-tailed duck population wintering in Europe, spend this time in the Baltic Sea [10]. North American studies also suggest a long term decline for long-tailed ducks [13]. Overall, the global trend for this species shows a decrease of around 50% over three generations (i.e. 27 years, 1993–2020) [4]. In response to those studies, the International Union for Conservation of Nature (IUCN) has reclassified the long-tailed duck from ‘Least Concern’ to ‘Vulnerable’ in 2012 [14].

Reasons for the severe decline are not fully understood, but include threats in the wintering areas and in the Arctic breeding grounds. In the Baltic Sea, one of the major threats is the entanglement in gillnets, killing around 90.000 individuals annually [15, 16]. Due to the ducks’ susceptibility to gillnet bycatch [7], around 1–5% of the total Baltic Sea population are lost every year [17]. Marine traffic is intense on the Baltic Sea, resulting in frequent small oil spills and infrequent large ones, which represent a further threat [4, 14]. Close to the breeding grounds, increasing exploitation of oil in the Arctic also contributes to the risk of oil spills [18]. Additionally, the time period of the wing moult (late July–early September) makes the ducks particularly susceptible to those threats, because the birds become flightless for 3–4 weeks [1, 3, 19, 20]. In most of their range, long-tailed ducks are also hunted [4, 21]. For example, nearly 20.000 individuals were reported as hunted in Finland in 2013 [4] and around 14.000 individuals per year were estimated hunted in Russia between 2013 and 2016 based on photos collected from hunters, but real numbers in Russia are suggested to be much higher [22]. In addition to direct impacts of human activities, indirect effects also affect long-tailed ducks. Climate change influences the life cycle of long-tailed ducks. Following the collapse of lemming cycles since 1995, presumably as a result of climate change, the predator pressure on nesting birds, their eggs, and offspring has probably increased [23, 24]. Climate change further leads to rising water temperatures, which changes the composition of phytoplankton communities, the basis of aquatic food webs. In consequence, changes may occur in the availability and quality of important food for long-tailed ducks, such as mussels [25] and other benthic invertebrates, which represent their major food source [26–28]. Additionally, long-tailed ducks are susceptible to avian influenza, avian botulism, and avian cholera, which can kill numerous birds in a short time [4, 29, 30].

To assess the importance of the widely scattered threats across the yearly cycle, tracking data can reveal spatiotemporal distribution patterns of birds. This allows evaluating the relative impact of different threats on the declining population. An important objective is the identification of areas of critical importance during the annual cycle, including sites outside the Baltic Sea. The first tracking study of long-tailed ducks in Europe (Žydelis 2009, 2010, 2013, available on https://www.movebank.org) tracked birds caught in the Baltic Sea itself. The present study is the first to track long-tailed ducks from their Russian Arctic breeding grounds. Here, we focus on the spatiotemporal pattern in the annual cycle of female long-tailed ducks equipped with geolocators in their Arctic breeding area on Kolguev Island. Specifically, we identify (1) staging areas, (2) moulting sites, (3) wintering areas, and (4) the phenology of these stages. Thereby, we fill current knowledge gaps of the movement ecology of long-tailed ducks, as described in the Agreement on the Conservation of African-Eurasian migratory waterbirds (AEWA)—single species action plan [4].

## Methods

### Capture of birds

Female long-tailed ducks (n = 48) were caught in the interior of Kolguev Island [69.138° N, 48.848° E] in the Barents Sea for deployment of light level geolocators between mid-June and beginning of August 2017. Until mid-July, birds were caught mainly with mist nets erected between small lakes in the tundra (n = 12). Later, unweighted gillnets were used on lakes to catch resting ducks (n = 8), ducks during chick-rearing (n = 2) or wing moult (n = 26). Birds were untangled immediately after capture to prevent drowning [31–33]. Only females were chosen because they show higher site fidelity, thus enabling recapture. Long-tailed ducks usually pair in the wintering grounds and on spring migration. Subsequently, males follow their partner to the breeding area [1]. Therefore, males are less suitable for deploying geolocators, as their return to the same breeding area is less predictable. We recovered 19 of the 48 deployed geolocators in the same area between mid-June and beginning of August 2018 (mean ± SD: 29 July ± 16 days) after 364 ± 11 days (range 356–403 days) of deployment. Most of the ducks were caught during wing moult (n = 16), but also during breeding season (n = 2), or resting on a lake (n = 1). After the recapture and removal of the geolocator, a new geolocator was attached to the bird for subsequent studies and the animals were released immediately. Additionally, nine tagged birds were identified but could not be recaptured. This corresponds to a recapture rate of 40% and a resighted rate of 58% birds in the study area.

### Geolocator attachment

Geolocators (Intigeo C-330, Migrate Technology Ltd, Cambridge, UK) were attached to numbered steel leg rings (Russian Ringing Centre, Moscow) using cable ties. The geolocators weigh 3.3 g each, representing a maximum of 0.6% of female body mass of 690 ± 56 g (range 580–810 g). This is well below the maximum recommended weight for tracking devices (3–5% of the body mass [34–37]). Geolocators recorded relative light level every minute and stored the maximum value every 5 min. Temperature was measured every 5 min and maximum, minimum and mean values were stored every four hours. The accuracy of temperature values for the logger was 0.5 °C. Water conductivity was recorded every 30 s on a relative scale between 0 and 127 and the maximum was stored every four hours. Loggers collected a wet/dry-state every 30 s. Wetness corresponds to the value 1 and dryness to 0, and values were summed up and stored every four hours on a relative scale between 0 and 480, reflecting the sampling rate of 30 s [38]. Ducks fitted with geolocators were additionally marked with nasal saddles, made from cattle ear tags and nylon fishing line or cable ties [39]. The nasal tag is necessary for identifying the bird for recapture, as the geolocator itself is nearly always invisible under field conditions.

### Geolocator data processing

Geolocators provide a maximum of two positions per day based on sunrise and sunset times. Sunset and sunrise events were assigned from relative light levels (IntiProc 1.03, Migrate Technology LtD, Cambridge, UK). All sunrise and sunset events were manually validated, and only unequivocal assignments were included in subsequent analyses. During the polar day, sunset and sunrise events were both assigned at the estimated midnight times.

In total we used four different location calculation approaches to display variability in methods and prevent overinterpretation. We used (I) GeoLight [40] with an individually calibrated sun elevation angle. Calibration was performed as rooftop calibration in Central Germany and sun elevation angle varied between − 5.0 and − 5.3. We used (II) GeoLight [40] with a fixed sun elevation angle of − 3.5, as used in [41, 42] and which was close to a mean angle of − 3.3 calculated for a Northern Hemisphere sea duck before [43]. The higher sun elevation angle was used, because rooftop calibration can result in more polewards estimates of latitude due to the lack of shading, compared to the attachment on the bird [44]. We used (III) GeoLight [40] with an individually calibrated sun elevation angle, using Hill-Ekstrom calibration from a stationary period in winter [45, 46], resulting in sun elevation angles between − 1.8 and − 9.8. We used (IV) the probGLS modelling process, which was optimised for seabirds and takes additional information from the geolocators into account. Those additional parameters are tagging/retrieval location and time, possible speed of the bird in flight or on water, and sea surface temperature. Additionally, the package can exclude the land area and areas covered by ice. Calibration of geolocators is not necessary for this method, as the model chooses the most likely sun elevation angle for each point individually [47]. To take the difference in salinity between freshwater, the mostly brackish Baltic Sea, and other marine habitats into account, we extended this model to include conductivity data. To do so, conductivity thresholds for the different water types were determined in temperature dependent saltwater solutions in the lab with an Intigeo C-330 logger. The probGLS R package has been updated to make this extension available (https://github.com/benjamin-merkel/probGLS). Data points during the equinox periods and polar day were included, as the model has an algorithm to account for missing latitude values. A table of model settings can be found in the Additional file 1.

Due to temporal overlap with equinox events and polar day, the post-moult and pre-breeding distributions (see definitions in next paragraph), as well as migrated distances, were calculated based on the probGLS models. To display spatial patterns and calculate core ranges during the post-moult and pre-breeding periods, we used kernel densities, calculated in R with the package adehabitatHR [48]. We used a generic grid of 100 cells and the ad hoc method for estimating the smoothing parameter.

### Definitions of staging periods

We characterized six staging periods within the annual cycle of the long-tailed ducks, based on water conductivity and longitudinal information (see Additional file 2). As most of the changes between life stages occur close to equinox events and during the polar day, we did not use latitudinal information for this purpose. The breeding stage (I) was defined as the time in which the bird does not enter salt or brackish water and does not show a change in longitudinal values. The post-moult or post-breeding stage (II) was defined as the time after the bird performs the first movement away from freshwater, indicated by a change in longitude, conductivity or both. Small-scale movements and changes in conductivity can occur during the post-moult stage. This ended when the bird started autumn migration (III), which was represented by a rapid change in longitudinal values. Autumn migration stopped and the wintering stage (IV) began when longitudinal changed by less than 10° and conductivity values remained constant. Subsequently, the onset of spring migration (V), noted as a further rapid change in longitudinal and conductivity values, defined the end of the wintering stage. Spring migration stopped when conductivity and longitudinal values (range ± 15° because inaccuracy is higher during the polar day) showed constancy again. The period following the spring migration was defined as pre-breeding stage (VI), and lasted until the bird stayed constantly in freshwater, and the breeding stage (I) started again. For all stages, we manually assigned the start and end time, the mean longitude, and the immersed water types (fresh, brackish, seawater or dry). Water types were defined via the conductivity values. Stopovers were noted during spring and autumn migration periods. Stopovers were defined as periods of at least two days within the migration stage without longitudinal changes.

**Fig. 1:**
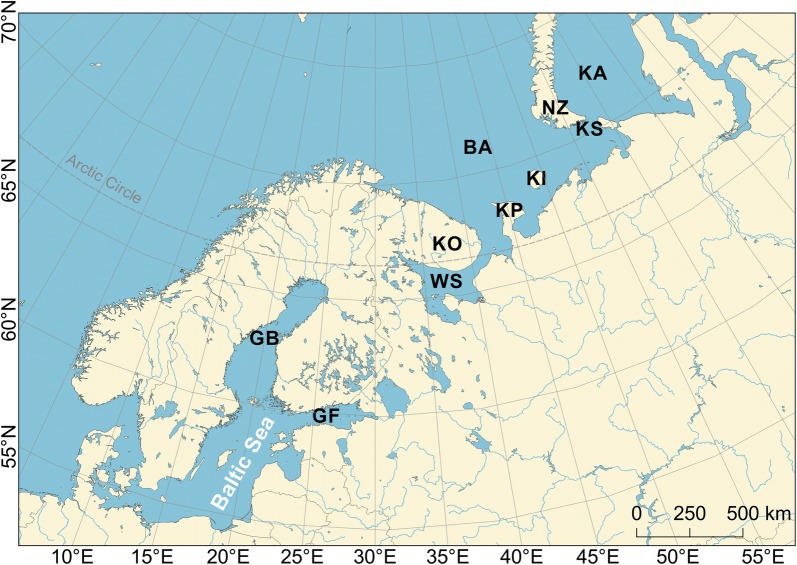
Map of locations mentioned in the text (BA—Barents Sea; GB—Gulf of Bothnia; GF—Gulf of Finland; KA—Kara Sea; KI—Kolguev Island; KO—Kola Peninsula; KP—Kanin Peninsula; KS—Kara Strait; NZ—Novaya Zemlya; WS—White Sea). The background map was provided by Natural Earth from their website https://www.naturalearthdata.com

Location names, mentioned in the text, are displayed in Fig. 1 and follow the nomenclature of HELCOM (Baltic Marine Environment Protection Commission) [49].

## Results

### Breeding stage

**Fig. 2:**
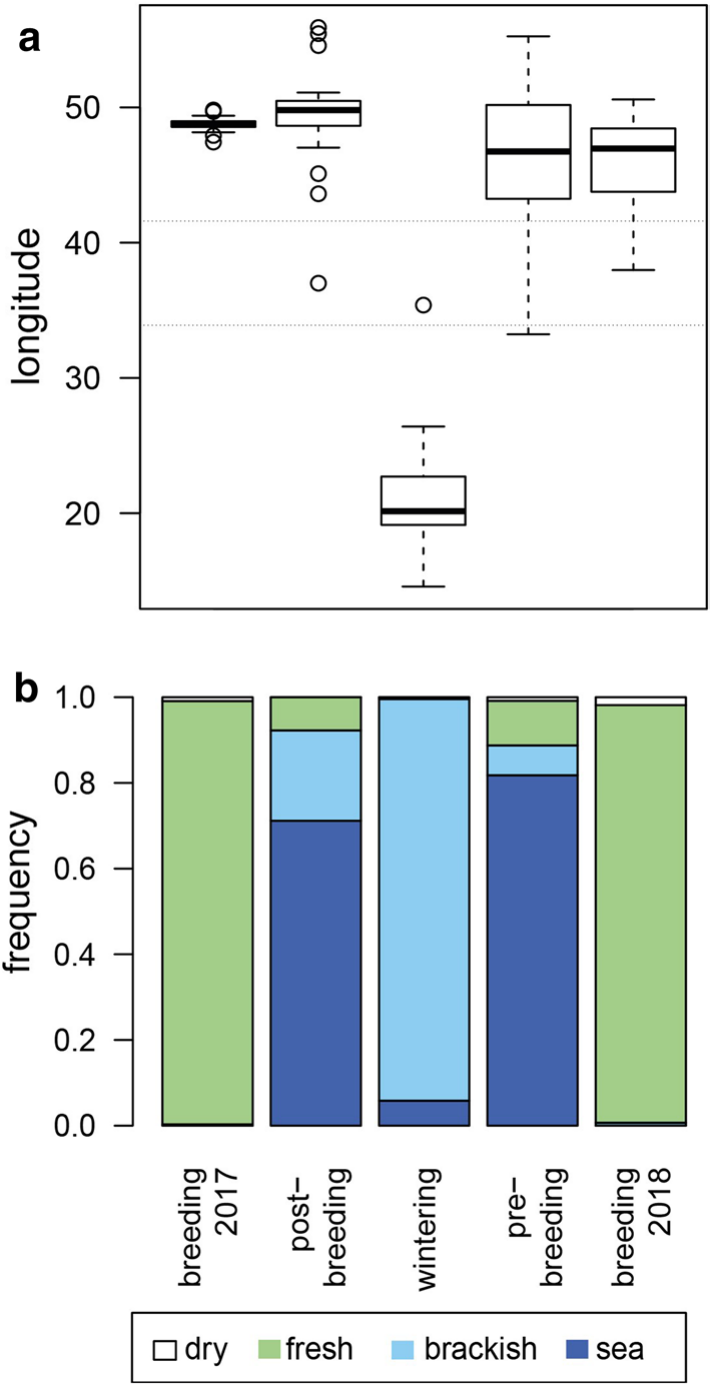
Longitudinal distribution (**a**) and water type usage (**b**) of female long-tailed ducks in the breeding stages of 2017 and 2018, the post-moult (or post-breeding), the wintering, and pre-breeding stage. Data are derived from a year-round tracking with geolocators. The pointed lines in **a** represent the longitudinal range of the White Sea between 33.9 and 41.6° E

In 2017, the breeding stage of the female long-tailed ducks ended mid-September, with a variation between end-August and mid-October and started in 2018 in mid-June, with a variation between beginning of June and beginning of July (detailed dates in Table 1). From these dates, we can estimate the duration of the breeding stage to be between 68 and 120 days (Table 1). The ducks spent their breeding stage at freshwater bodies (Fig. 2), as inferred from the definition of the staging periods. There was no evidence for detectable movements within the breeding stage with the tracking method using geolocators.

**Table 1:** Phenology of the stages of the annual cycle of female long-tailed ducks breeding on Kolguev Island, derived from one year of geolocator data between Summer 2017 and Summer 2018

Characteristic of life stage event	Mean ± SD	Range	n
Longitude late breeding stage 2017	49 ± 1° E	47–50° E	19
Timeframe post-moult stage			
Start	12 Sep. ± 11 days	29 Aug.–07 Oct.	19
End	15 Oct. ± 9 days	28 Sep.–01 Nov.	19
Duration post-moult stage	33 ± 10 days	19–50 days	19
Longitude post-moult stage	49 ± 4° E	37–56° E	19
Duration autumn migration	3 ± 3 days	0–11 days	19
Estimated distance autumn migration	1247 ± 320 km	519–1880 km	19
Timeframe wintering stage			
Start	17 Oct. ± 7 days	02 Oct.–01 Nov.	19
End	17 May ± 5 days	10 May–02 June	17
Duration wintering stage	212 ± 3 days	197–229 days	17
Longitude wintering stage	21 ± 4° E	15–35° E	19
Latitude wintering stage	59 ± 2° N	56–66° N	19
Duration spring migration^a^	2 ± 2 days	1–9 days	17
Estimated distance spring migration^a^	1273 ± 307 km	549–1713 km	15
Timeframe pre-breeding stage			
Start^a^	20 May ± 5 days	14 May–03 June	17
End	12 June ± 9 days	01 June–02 July	17
Duration pre-breeding stage^a^	23 ± 7 days	13–34 days	17
Longitude pre-breeding stage^a^	46 ± 6° E	33–55° E	17
Longitude early breeding stage 2018^a^	46 ± 3° E	37–50° E	17
Duration breeding stage (estimated)	91 ± 13 days	68–120 days	17

The estimates of migration distance are based on locations, modelled with probGLS

^a^Longitudes and separation of life stages estimated from light curves of 24 h daylight

### Post-moult stage

**Fig. 3:**
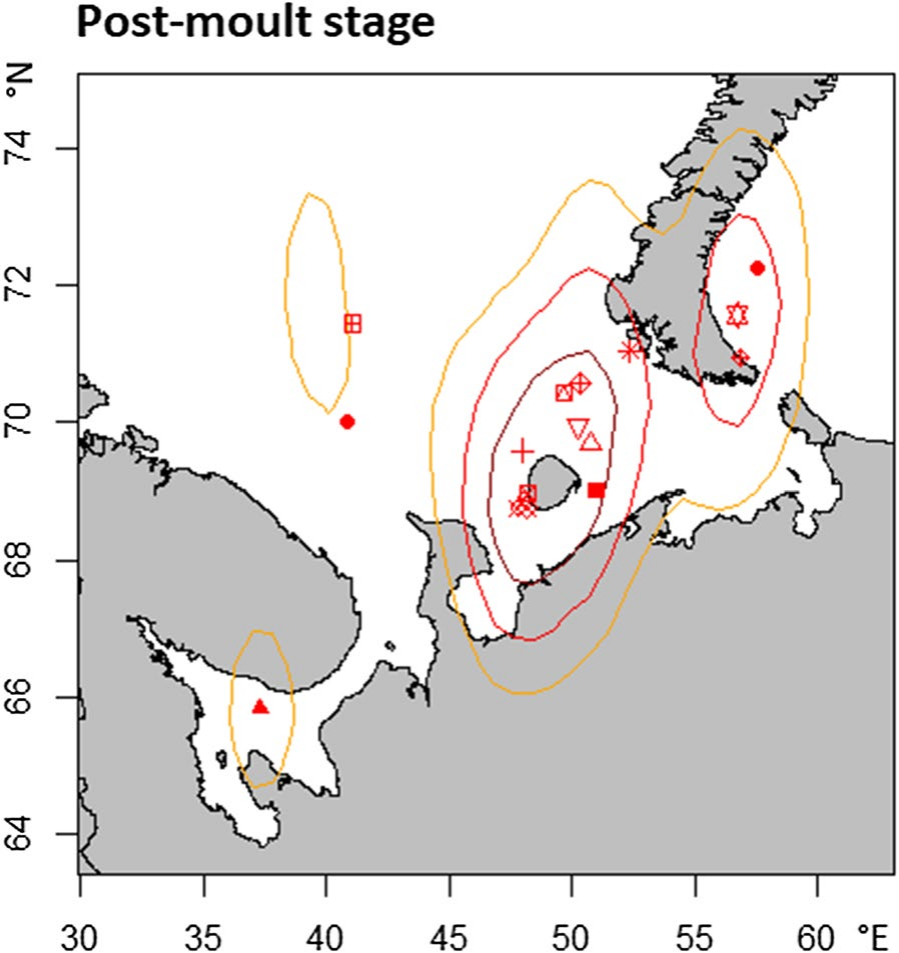
Distribution of female long-tailed ducks during the post-moult (or post-breeding) stage. Dark red, red and yellow areas represent 25, 50 and 75% kernel utilisation densities, respectively. Symbols represent individual median centroids. Due to the equinox period, only points, located at sea could be used (see “Methods” section; probGLS). The map was obtained from the R-package “maps”

After moving away from the breeding grounds, the ducks underwent a post-moult (or post-breeding) stage primarily between mid-September and mid-October for 33 ± 10 days (Table 1). Of the 19 tracked birds, 16 spent time at sea in the post-moult stage. Of these, 11 birds stayed exclusively at sea, whereas others performed additional movements towards brackish or fresh water. None of the individuals stayed exclusively on freshwater lakes during the post-moult stage (see Additional file 2). Of the 16 individuals that stayed at sea in the post-moult stage, 11 used the Barents Sea around Kolguev Island (Fig. 3), six of them exclusively. The remaining five long-tailed ducks dispersed north, east and west and used the Barents Sea or the Kara Sea near Novaya Zemlya Archipelago and the White Sea in the post-moult stage (Fig. 3). The three individuals that did not spend time in salt water in this stage showed no longitudinal movement from the breeding site before starting autumn migration. Locations for these birds cannot be calculated because latitudinal information is missing due to equinox and the lack of sea surface temperature as additional information for the probGLS model.

### Autumn migration

Most long-tailed ducks performed a rapid movement towards their wintering ground with a mean duration of autumn migration of 3 ± 3 days and a distance of 1247 ± 320 km (Table 1). Four birds performed stopovers at the longitudes 35, 39, 38 and 38° E and for 4, 7, 6, and 3 days, respectively. When excluding these birds, the mean duration of autumn migration was 1.6 ± 1 days and mean distance covered was 1209 ± 317 km. Although the autumn migration period is close to the autumn equinox, the probGLS model calculated locations for these stopovers: the first three were in the Barents Sea far from the Russian coast, and the last was in the White Sea.

### Wintering stage

**Fig. 4:**
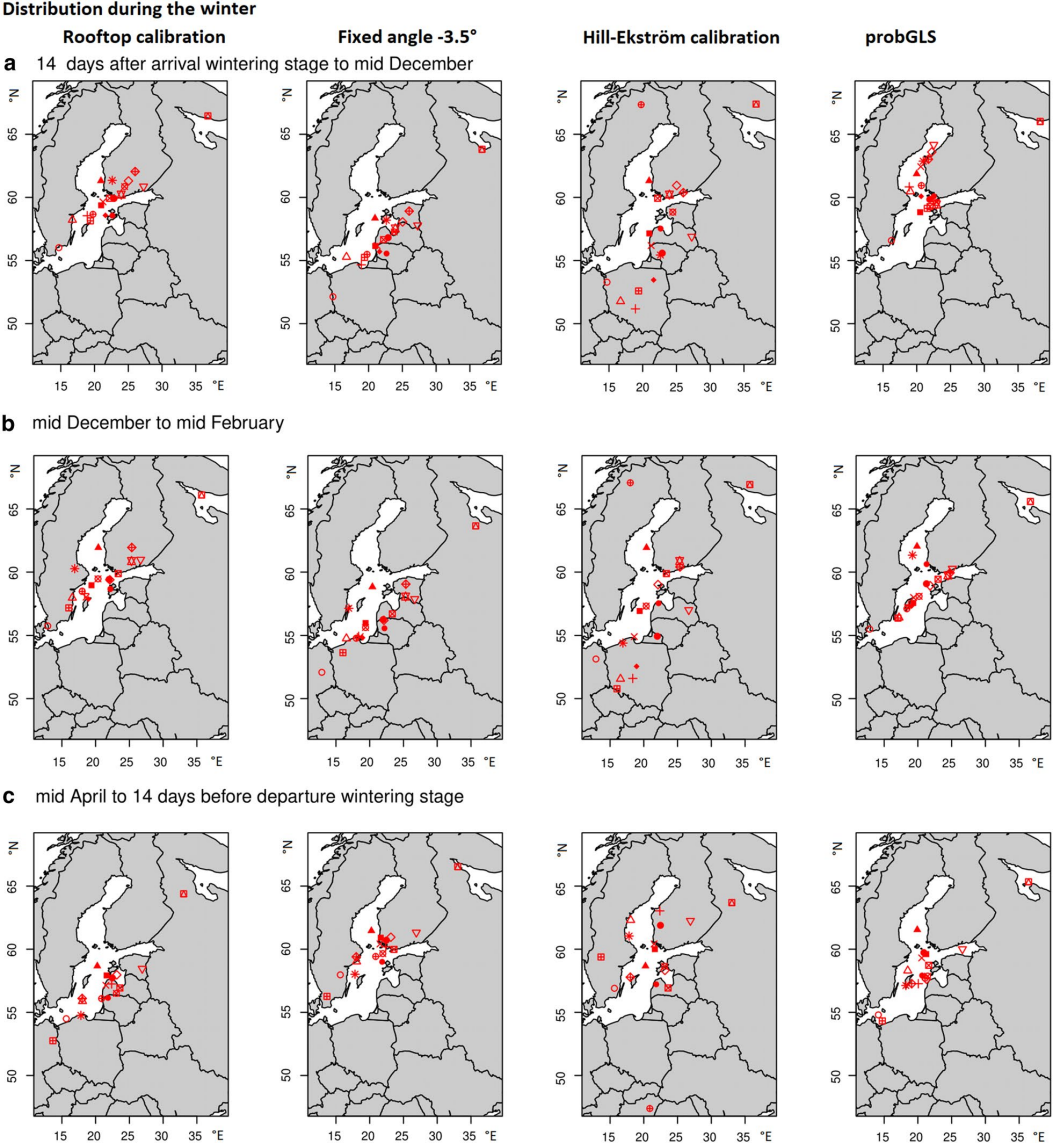
Distribution of female long-tailed ducks during the wintering stage in three different timeframes (**a**–**c**), calculated with four different models. Symbols represent individual median centroids. The map was obtained from the R-package “maps”

The long-tailed ducks stayed at their wintering sites between mid-October and mid-May for 212 ± 3 days (Table 1). Of the 19 tracked long-tailed ducks, 18 used the Baltic Sea as wintering ground, while one individual used the White Sea (Fig. 4). The variation in locations is caused by differences among individuals rather than movements of individual birds, as individuals showed preferences for relatively distinct regions (Fig. 4 and Additional file 3).

### Spring migration and pre-breeding stage

**Fig. 5:**
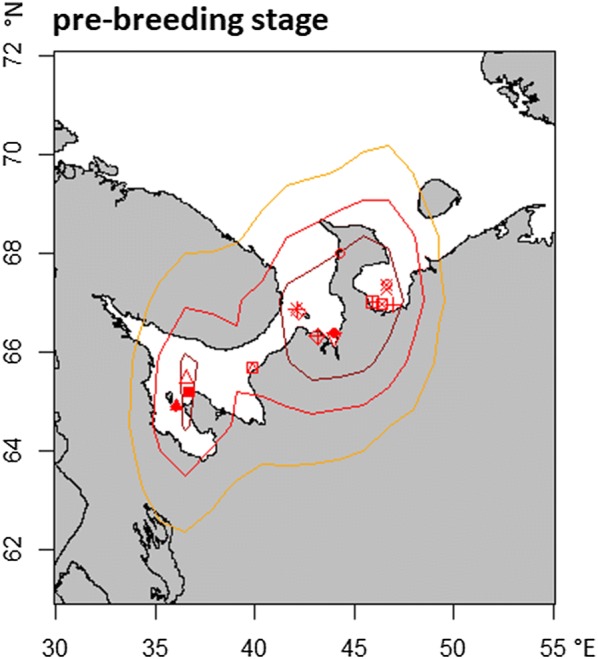
Distribution of female long-tailed ducks during the pre-breeding stage. Dark red, red and yellow areas represent 25, 50 and 75% kernel utilisation densities, respectively. Symbols represent individual median centroids. Due to the polar day, only points, located at sea could be used (see “Methods” section; probGLS). The map was obtained from the R-package “maps”

Spring migration lasted 2 ± 2 days and distance travelled was 1273 ± 307 km (Table 1). Stopovers were not recorded, but the transition to the pre-breeding stage was difficult to assess. This is caused by inaccurate longitude values in this period due to the start of polar day. During this pre-breeding stage, all birds spent most of their time on seawater (Fig. 2) in coastal areas between the White Sea, the Kanin Peninsula and their breeding ground Kolguev Island (Fig. 5). Movements to brackish water largely occur towards the end of the pre-breeding stage (see Additional file 2). Only one bird spent the pre-breeding time mainly on freshwater with short trips to the sea in the very beginning and end of its pre-breeding stage at a mean longitude of 52 ± 12° E.

## Discussion

Knowledge about movements of migratory species is crucial to develop and successfully implement conservation action, especially for species that live in remote areas and migrate over considerable distances, like long-tailed ducks. In line with this, the present study revealed, for the first time, year-round spatiotemporal patterns of long-tailed ducks starting at one of their European breeding grounds.

After the wing moult period at freshwater bodies, most female long-tailed ducks from Kolguev Island moved to the sea, where they spent approximately one month. Afterwards, they performed a rapid autumn migration towards their wintering ground in the Baltic Sea (n = 18) or the White Sea (n = 1). Spring migration mirrored the rapid movement of autumn migration. Before they returned to their freshwater breeding grounds, the birds stayed mainly at sea for about three weeks.

### Breeding stage and wing moult

Long-tailed ducks have a very complicated and unique moult scheme and moult different parts of their plumage at different times of the year [1, 50–52]. The replacement of the wing and tail feathers needs many resources and makes the ducks flightless. Consequently, this period (late July–early September) is the most sensitive part of the moult scheme and further considered as “the moult”.

In contrast to other studies on sea ducks [53–56], we cannot distinguish between moulting and breeding period or identify moult migration, as we observed no extensive movements or change in water type in the corresponding timeframe between end of July–beginning of September in 2017 [1, 3, 19, 20]. Of the 19 ducks, 12 were initially caught during moult itself at freshwater lakes and one during chick-rearing. Of the remaining six individuals that also stayed at freshwater lakes for moult, three were already caught in June. According to these observations, these females moulted at locations that are not spatially distinguishable from the breeding site with geolocators (ca. < 200 km), most likely on Kolguev Island. Moult at tundra lakes is known for female long-tailed ducks, as described in [57–59]. In a North American tracking study, moult within the breeding area was observed in three out of eight female long-tailed ducks, although moult migration of the same individuals had been recorded the year before [55]. However, moult migrations ranging from a few to several hundred kilometres were described for female long-tailed ducks [60]. Major moulting areas in the European Russian Arctic were recorded in several bays along the mainland coast and on the Barents Sea shelf [61]. Furthermore, ducks that were not resighted in 2018 could have performed moult migration in 2018, even if they were caught during moult on freshwater lakes in 2017.

### Post-moult stage

The 19 tracked long-tailed ducks spent their post-moult or post-breeding stage mainly at sea, beginning in mid-September on average and lasting for about one month, before autumn migration was initiated in mid-October. The start date of the post-moult stage (Table 1) was consistent with the end of the moulting period described for satellite-tracked female long-tailed ducks in Alaska [60] and with long-tailed ducks moulting on the Kara Sea [62]. For that stage, they mainly moved to the closest coastal waters around Kolguev Island or dispersed to north, east or west (Fig. 3). We observed that some birds spent this time also on fresh and brackish water, most likely estuaries, lagoons or other waters near the coast. Interestingly, four individuals moved to the Kara Sea or eastern part of the Barents Sea for the post-moult stage, which is in the opposite direction from their autumn migration route. A post-moult movement against the autumn migration direction was also observed in one of the three individuals tracked by Mallory et al. [63]. Similar to our study, Petersen et al. also observed a pronounced post-moult stage of female long-tailed ducks from Southeast Alaska [60]. In contrast to that, Bartzen et al. did not observe a post-moult stage near the moulting or breeding site, but described multiple stages on autumn migration, lasting between two weeks and one month [55]. The main difference between the European and North American populations is that birds wintering in the Baltic Sea have to cross a land barrier. We suggest that the post-moult stage is connected with autumn migration and is differently pronounced, depending on the geographical arrangement of coastlines and land barriers en route. In any case, the post-moult staging areas may be important for accumulating energy required for autumn migration, as suggested by carcass analyses [64].

### Autumn migration

Most long-tailed ducks in our study showed a rapid movement from their post-moult area into the wintering ground without extensive stopovers. As mentioned above, autumn migration in long-tailed ducks can vary between a rapid migration and a slow movement with multiple stopovers, probably depending on the geography.

The passage through the White Sea is suggested to be the main flyway for the western Russian long-tailed-duck [65, 66]. In our study, four of 18 birds performed stopovers at the longitude of the White Sea. Additionally, three individuals spent their post-moult stage at the longitude of the White Sea (Fig. 3). The timeframe of autumn migration previously observed in the White Sea considerably overlaps with the autumn migration period deduced from our tracking data [67]. After crossing the White Sea, birds wintering in the Baltic Sea have to fly overland, either via the Gulf of Bothnia, or through the Gulf of Finland. Depending on the used method for winter distribution, our data represent both routes, as also suggested by Mathiasson [68].

### Wintering stage

All tracked female long-tailed ducks, except one individual, spent their winter in the Baltic Sea. This is similar to the estimation that 90% of the European overwintering long-tailed ducks winter in the Baltic Sea [10]. The White Sea, where one bird spent its winter, is also known as a wintering ground for long-tailed ducks in moderate numbers [59, 65, 69–72]. Wintering long-tailed ducks are also reported from several places in the Barents Sea, but numbers are generally low [73–77]. Interestingly, individual birds used different areas within the Baltic Sea during winter, although they were all caught within a small area on Kolguev Island (Fig. 4 and Additional file 3). This suggests no fine scale association between breeding and wintering areas, but a broad-scale connectivity between the Baltic Sea and birds breeding on Kolguev Island. Petersen et al. made a similar observation for tracked female long-tailed ducks from the Yukon Delta and concluded that wintering populations may contain birds from several breeding populations [60]. The same applies to Steller’s eiders (*Polysticta stelleri*) tracked from breeding grounds in Alaska [78] but is in contrast to common eiders (*Somateria mollissima*) from Arctic Canada, where breeding populations segregate in the wintering grounds [56, 79]. On the flip side, long-tailed ducks that were tracked from a single wintering area in the Baltic Sea had very distinct breeding areas (Žydelis 2009, 2010, 2013, available on https://www.movebank.org).

The observed central wintering areas (Fig. 4 and Additional file 3) largely overlap with the already known core areas for sea ducks in the Baltic Sea [1, 2, 80–82]: the coastal areas and off-shore banks between Denmark and the southern coasts of Finland.

The duration of the wintering stage in our study was 212 ± 8 days (Table 1), or 211 ± 7 days, when excluding the bird that wintered in the White Sea. This was considerably longer than in a tracking study of North American long-tailed ducks, with 155 ± 22 days [55], but the definition of stages differed slightly. Wintering periods of other sea duck species are also shorter (surf scoters (*Melanitta perspicillata*): 133 days [83], white-winged scoters (*Melanitta deglandi*): 189 days [84], common eiders: 141 ± 10 and 128 ± 3 days [85], black scoters (*Melanitta americana*): 147 ± 4 days [86], king eiders (*Somateria spectabilis*): 160 ± 68 days (range: 54–294) [53]). The long period of around 58% of the year that our long-tailed ducks spent in the Baltic Sea, stresses the importance of this wintering ground for the species. For males, the wintering period could be even longer, as males are known to migrate around a week earlier in autumn than females and juveniles [87].

### Spring migration and pre-breeding period

The observed time of departure from the Baltic Sea (17 May ± 5 days) overlaps considerably with the time of peak migration of long-tailed duck and common scoter (*Melanitta nigra*) observed in southern Finland between 10 and 28 May in the years 1960–1962 [88]. In addition, long-tailed ducks that were tracked from the south-western Baltic Sea with satellite transmitters left the Baltic Sea in mid-May (Žydelis 2009, 2010, 2013, available on https://www.movebank.org).

Our data include the spring migration route towards the White Sea, also reported by Lapshin et al. [89]. After migration over the Finnish mainland, most birds made stops on the White Sea itself and at coastal areas of the Barents Sea between Kolguev Island, the Kanin Peninsula and the Kola Peninsula. This resembles the descriptions in Mineyev and Mineyev & Mineyev of a main migration route along the coastline [73, 90]. Furthermore, they describe a movement along river valleys of the Pechora River and its branches to reach the breeding grounds. Our data are in line with such a further movement in river valleys, as at least five birds spent the end of the pre-breeding stage in brackish water, indicating estuaries. Long-tailed ducks tracked by Žydelis (2009, 2010, 2013, available on https://www.movebank.org) showed a similar spring migration and pre-breeding pattern with a stop in the White Sea and further stops on the sea closer to their breeding ground [2]. In addition, the long-tailed duck spring migration from the Great Lakes in North America shows similarities. Those birds performed a series of rapid movements separated by relatively long stopovers at coastal sites [55]. Because we observed these ‘long stopovers’ close to the breeding area, we defined them as pre-breeding stage with a short movement to the breeding ground afterwards, rather than spring migration with stopovers. In summary, coastal areas on the migration route provide important resting sites for long-tailed ducks, especially as they spend around three weeks at sea before heading inland towards the breeding ground. Long-tailed ducks breeding further east might show spring staging areas along the coastline of the Kara Sea. The date of arrival at the breeding ground between early June and July overlaps with descriptions in the literature for long-tailed ducks breeding in the European Russian tundra [73, 74, 91].

### Assessment of threats

The Baltic Sea represents an important staging area for long-tailed ducks where the birds in this study spent as much as 58% of the year. This stresses the significance of threats in the Baltic Sea, like gillnet bycatch. Conservation measures, including implementation of marine protected areas, testing of sea bird friendly fishing gear and continued monitoring, should therefore persist or be enhanced, especially as threats and distribution are well known (see “Background” section). Together with the older tracking data of Žydelis (2009, 2010, 2013, available on https://www.movebank.org), our data show that the distribution of long-tailed ducks in the Baltic Sea likely does not reflect specific breeding areas, i.e., there is no fine scale migratory connectivity. Observed declining numbers of wintering long-tailed ducks or declining proportions of juveniles at specific sites in the Baltic Sea can therefore not give us any indication for spatial distribution of problems in the breeding ground. Conservation management should therefore treat this West Siberia/North Europe population as a whole [4]. We identified the Baltic Sea as the dominant wintering ground of long-tailed ducks breeding on Kolguev Island. Thus, most of the birds had to cross Finland or even wintered there, while hunting bags of these birds are still high. The Birds Directive of the European Union (EU) might be a suitable tool to address this in Finland, whereas EU legislation does not apply to Russia. The re-categorisation from Annex II to Annex I of the EU Birds Directive might be an effective tool for conservation in Europe, i.e., in the Baltic Sea region, not only in terms of hunting, but because member states have to implement “special conservation measures” for those species [92]. We showed that the seas around the breeding ground, specifically the coastal areas of the Barents Sea, Kara Sea and White Sea, are staging areas, where the birds spent around two months of the year. Additionally, many long-tailed ducks might use the sea for moulting [55, 60, 77, 93], especially males, but our dataset was limited to females that stayed in the tundra for moult. Due to climate change and the resulting reduced ice cover, anthropogenic pressure will increase in the aforementioned seas. One of these pressures on the ecosystem will be increased marine traffic in the Northeast Passage [94]. The proposed route crosses the Kara Strait [94] and therefore coastal areas that we identified as post-moult areas. The most interesting time for marine traffic with the least ice cover is September. This considerably overlaps temporally with the post-moult stage. Ship traffic results in loss of foraging time and increased energy expenditure [95, 96]. Illegal discharges of oil or accidental oil spills are threats accompanying increased ship traffic and are already among the currently recognised problems in the Baltic Sea. Additionally, the exploitation of oil and gas in the Russian Arctic has intensified and contributes to the risk of oil spills [18]. A further expected result of climate change is an increase of fisheries in the high latitude oceans, potentially aggravating the threat of sea duck bycatch, due to entanglement in gill nets, especially in coastal areas and offshore banks [97]. Moreover, the pronounced climate change in the Arctic regions will also affect ecological aspects of these stopover sites, specifically the composition and quality of food, including bivalves. Because the post-moult stage is important for providing energy for autumn migration [64], carry-over effects on survival during migration are likely. Further, we do not know what carry-over effects the pre-breeding stage might have on breeding success. A linkage, including carry-over effects, between those stages and with the wintering stage is likely, as the lakes at the breeding habitat do not provide a lot of food during arrival [1]. In summary, the well-known threats of the Baltic wintering ground could extend to high latitude oceans in the near future.

## Conclusions

Our geolocator tracking results of female long-tailed ducks from Kolguev Island agree with many observations and studies from several sites en route. Thus, we managed to connect and validate information from scattered and partly unheeded, non-English publications. Consequently, we are able to draw a complete picture of the annual spatiotemporal distribution pattern of a representative number of long-tailed ducks. Of 19 birds tracked in this study, 18 used the Baltic Sea as their wintering ground. The wintering stage spans more than half of the year and therefore represents a crucial part of the annual cycle. Additionally, we identified an extensive post-moult and pre-breeding stage, during which ducks used the high-latitude seas along the north-western coast of Russia. This highlights the importance of these seas, and particularly their coastal areas, for the conservation of long-tailed ducks, especially in light of the anticipated increase in industrialization, fishing and marine traffic in the Arctic. To draw more comprehensive conclusions about the European wintering population, we suggest further tracking of individuals from other breeding sites in the Russian Arctic and from multiple years to investigate inter-annual variability and the movement ecology of males.

## Supplementary information

**Additional file 1.** Model parameters probGLS. The table shows the model parameters used in the probGLS modelling approach to calculate locations from geolocator data.

**Additional file 2.** Figure with 19 individual longitude/conductivity plots. The titles of the graphs represent the individual IDs of the long-tailed ducks. Black dots represent the longitude, calculated with the rooftop calibration method and including the polar day with 24 h daylight (see methods section). The black dashed lines indicate the longitudinal range of the White Sea between 33.9 and 41.6° E. Red dots show relative water conductivity values on a scale between 0 and 127. The red dashed line indicates the threshold between brackish and seawater. The timeframe is set between capture of the bird in 2017 and recapture in 2018.

**Additional file 3.** Figure with 19 individual winter distributions. Individual monthly distribution of 19 long tailed ducks (with ID), calculated with 4 different methods. The crosses represent the median centroid location in November (light blue), December (dark blue), January (purple), February (red), March (yellow, only for probGLS due to equinox) and April (black, missing for BG914 and BG065 due to tag failure in March). The map was obtained from the R-package “maps”.

## Data Availability

The datasets supporting the conclusions of this article are deposited in the movebank.org repository under the study name “Long-tailed ducks GLS 2018”.
