# Supplementary material for: Year-round spatiotemporal distribution pattern of a threatened sea duck species breeding on Kolguev Island, south-eastern Barents Sea
Source: BMC Ecol. 2020 May 25;20:31. doi: 10.1186/s12898-020-00299-2 (PMC7249297; doi:10.1186/s12898-020-00299-2)
Supplement: Supplementary file 1 — Additional file 1. Model parameters probGLS. The table shows the model parameters used in the probGLS modelling approach to calculate locations from geolocator data. [file 12898_2020_299_MOESM1_ESM.pdf]

Karwinkel et al. “Year-round spatiotemporal distribution pattern of a threatened sea duck species breeding on Kolguev Island, south-eastern Barents Sea”

Additional file 1: Table S1. Model parameters for sea duck geolocation data proceeding used in the function prob_algorithm_exp of the R package probGLS.

model parameter	values used
tagging.date<br>retrieval.date<br>tagging.location	individually for each bird
particle.number	1000
iteration.number	100
boundary.box	20° W to 80° E for longitude<br>50° N to 80° N for latitude
days.around.fall.equinox	14 / 21
days.around.spring.equinox	21 / 14
range.solar	-7° / -1°
speed.dry	14 / 7 / 23 m/s
speed.wet	0 / 0.5 / 2.6 m/s
sst.sd	0.5 °C (accuracy of the logger)
max.sst.diff	3 (default)
ice.conc.cutoff	95 %
wetdry.resolution	30 s (sample rate of the logger)
cond.threshold	115 / 75 (upper, lower)
