# Supplementary figures and images for: Year-round spatiotemporal distribution pattern of a threatened sea duck species breeding on Kolguev Island, south-eastern Barents Sea
Source: BMC Ecol. 2020 May 25;20:31. doi: 10.1186/s12898-020-00299-2 (PMC7249297; doi:10.1186/s12898-020-00299-2)

Additional file 2:

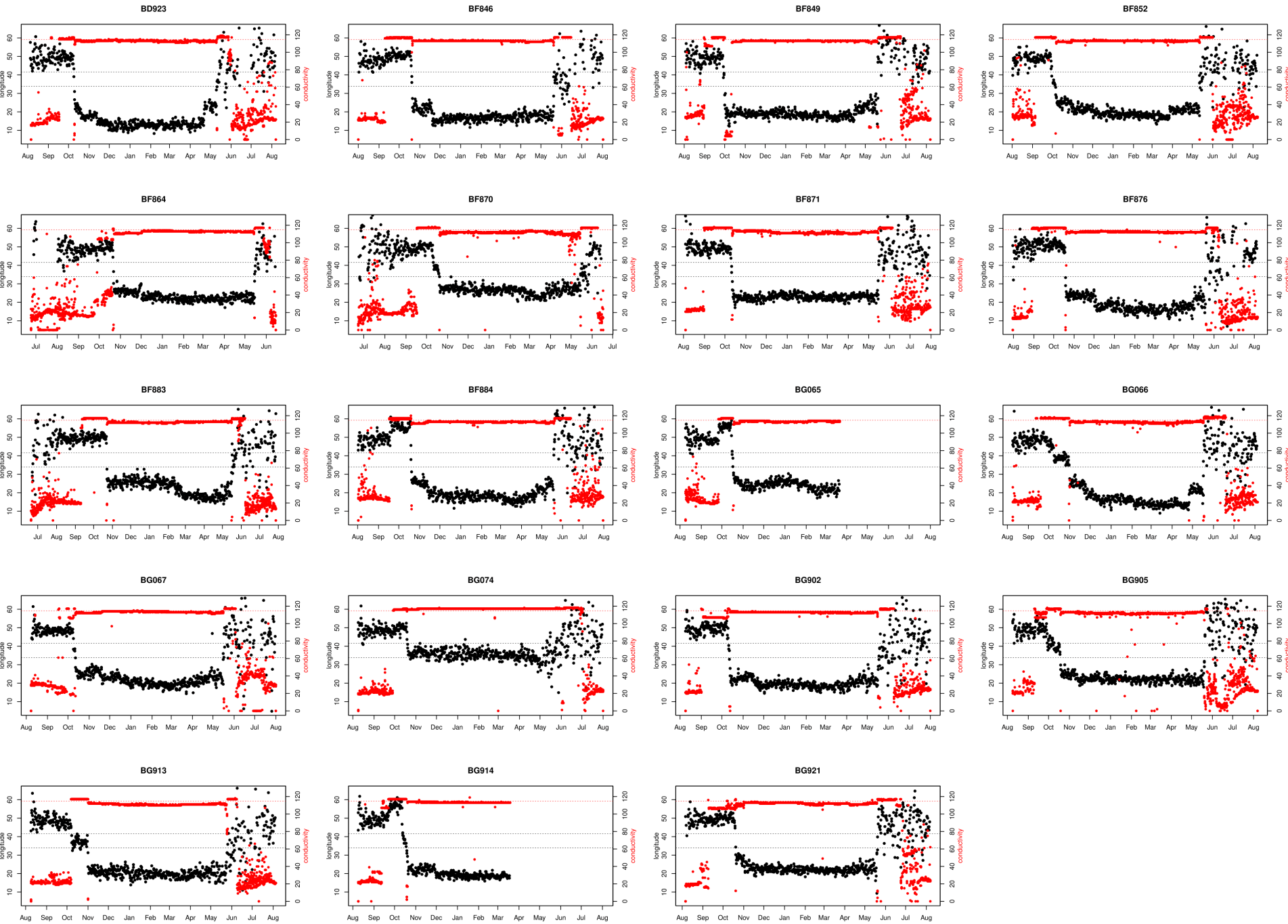

Supplement: Supplementary file 2 — Additional file 2. Figure with 19 individual longitude/conductivity plots. The titles of the graphs represent the individual IDs of the long-tailed ducks. Black dots represent the longitude, calculated with the rooftop calibration method and including the polar day with 24 h daylight (see methods section). The black dashed lines indicate the longitudinal range of the White Sea between 33.9 and 41.6° E. Red dots show relative water conductivity values on a scale between 0 and 127. The red dashed line indicates the threshold between brackish and seawater. The timeframe is set between capture of the bird in 2017 and recapture in 2018. [file 12898_2020_299_MOESM2_ESM.pdf]
