# Supplementary material for: Year-round spatiotemporal distribution pattern of a threatened sea duck species breeding on Kolguev Island, south-eastern Barents Sea
Source: BMC Ecol. 2020 May 25;20:31. doi: 10.1186/s12898-020-00299-2 (PMC7249297; doi:10.1186/s12898-020-00299-2)
Supplement: Supplementary file 3 — Additional file 3. Figure with 19 individual winter distributions. Individual monthly distribution of 19 long tailed ducks (with ID), calculated with 4 different methods. The crosses represent the median centroid location in November (light blue), December (dark blue), January (purple), February (red), March (yellow, only for probGLS due to equinox) and April (black, missing for BG914 and BG065 due to tag failure in March). The map was obtained from the R-package “maps”. [file 12898_2020_299_MOESM3_ESM.pdf]

Karwinkel et al. "Year-round spatiotemporal distribution pattern of a threatened sea duck species breeding on Kolguev Island, south-eastern Barents Sea"

Additional file 3
Model outputs for the winter distribution of long-tailed ducks with four different modelling methods.

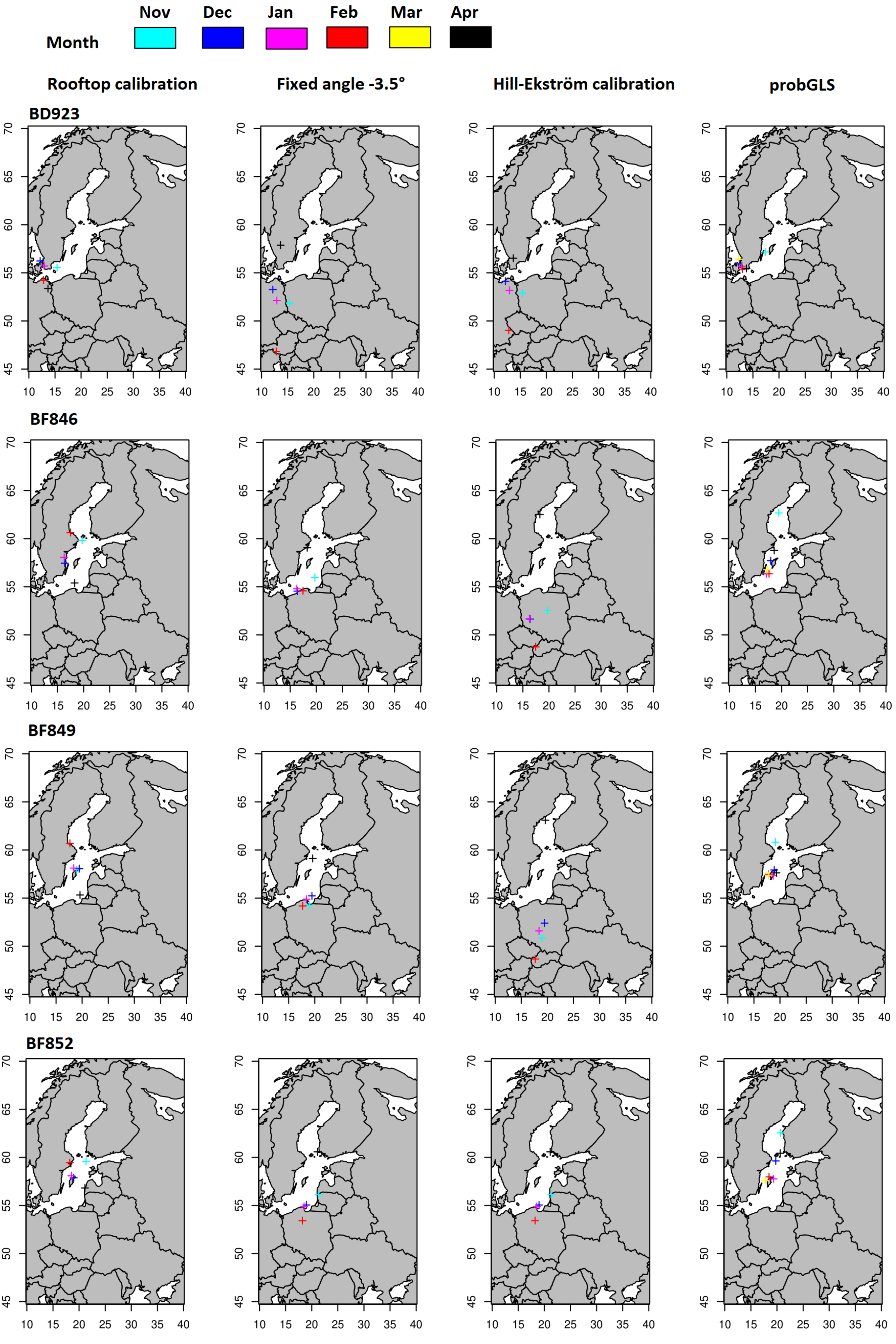

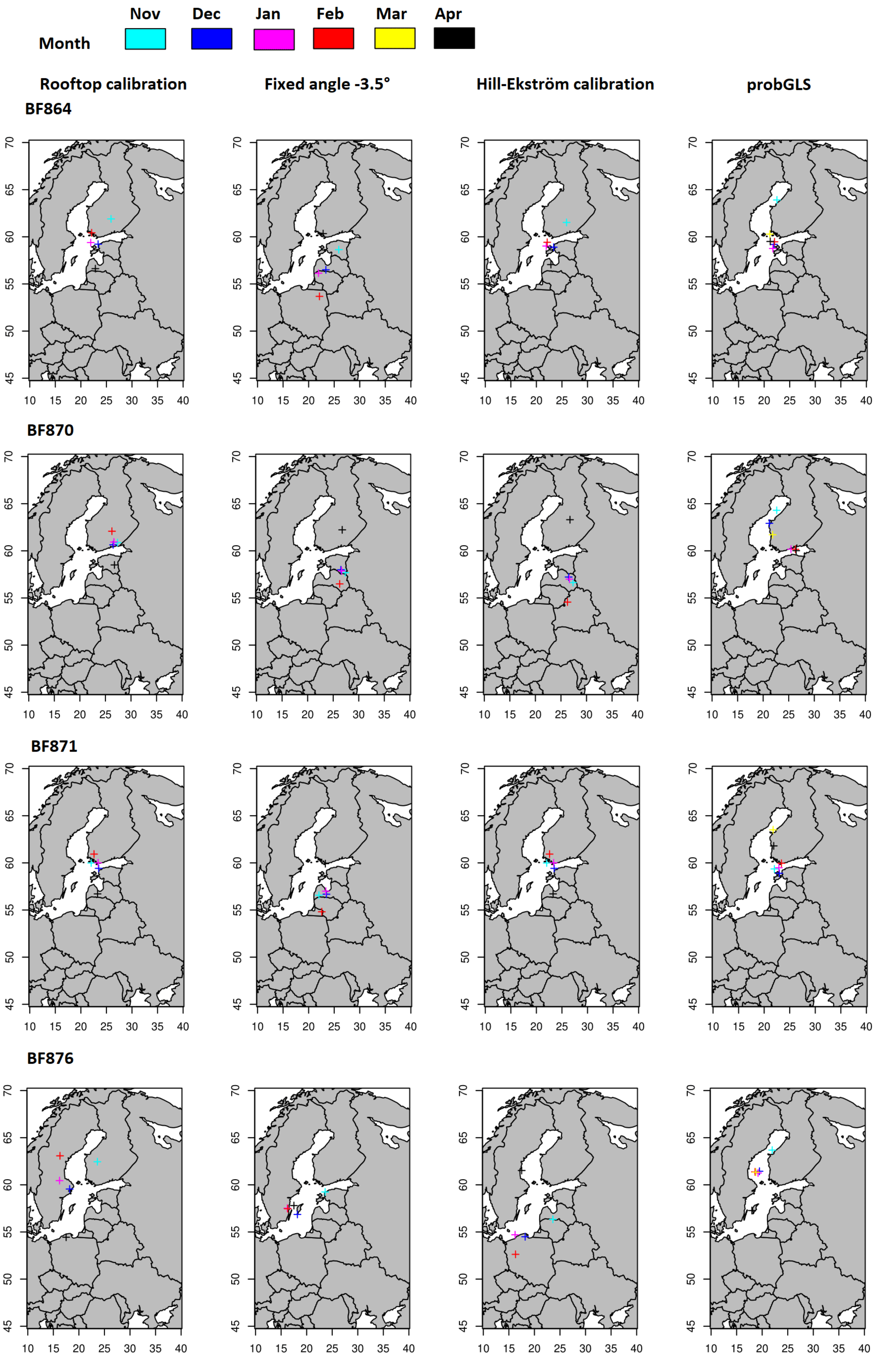

Month
Nov
Dec
Jan
Feb
Mar
Apr
Rooftop calibration
Fixed angle -3.5°
Hill-Ekström calibration
probGLS
BF864
BF870
BF871
BF876

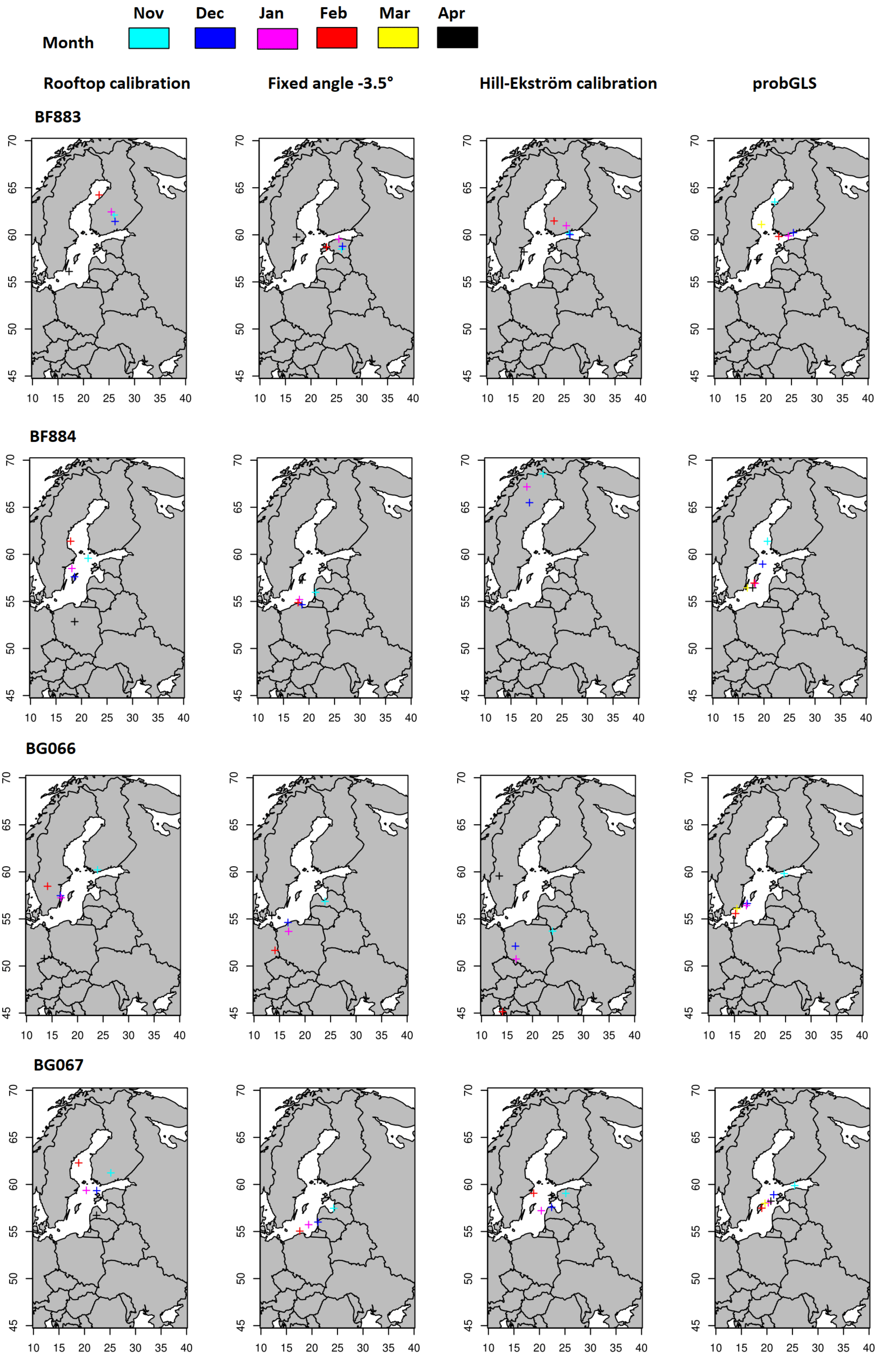
Month
Nov
Dec
Jan
Feb
Mar
Apr
Rooftop calibration
Fixed angle -3.5°
Hill-Ekström calibration
probGLS
BF883
BF884
BG066
BG067

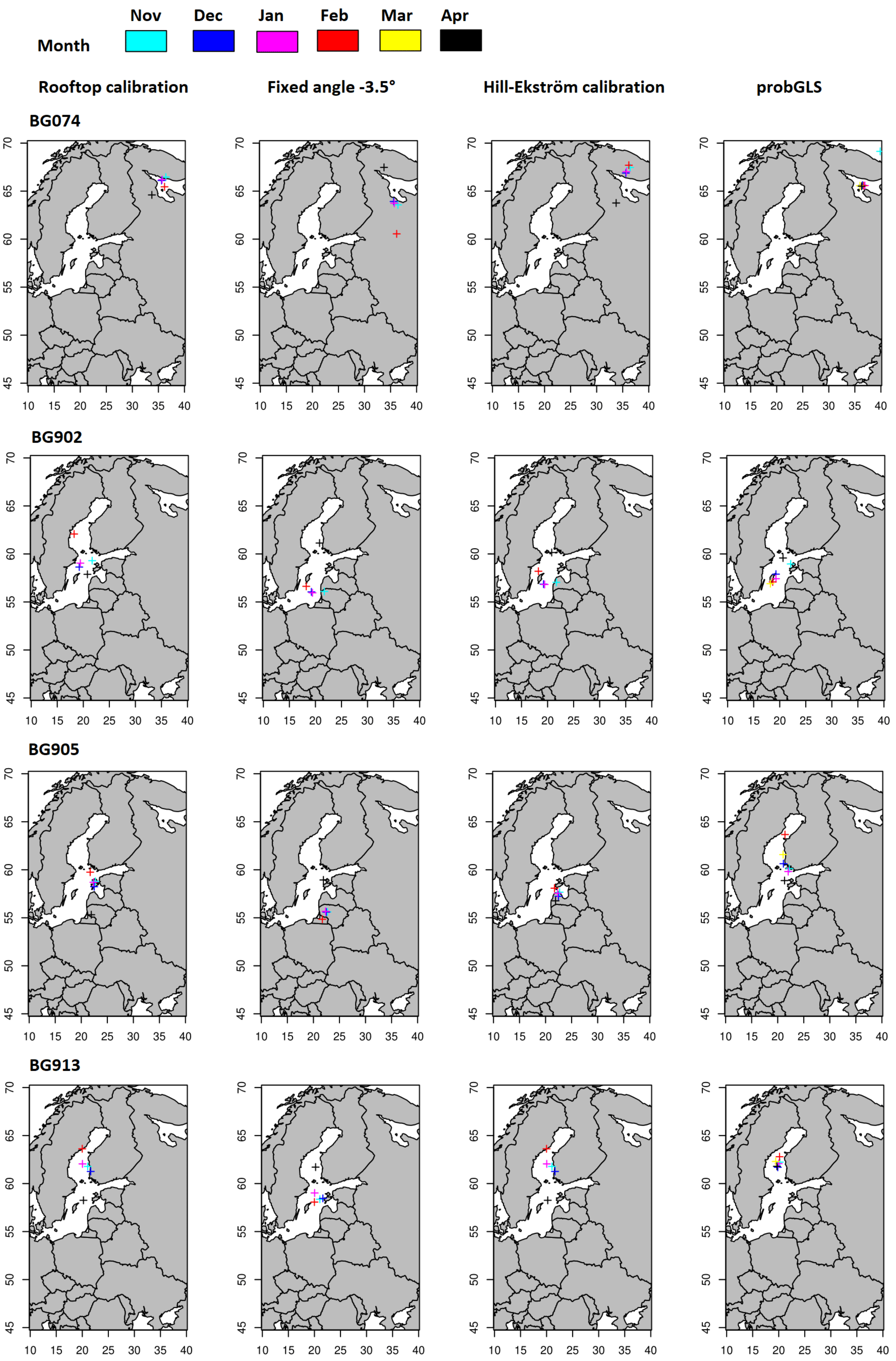
Month
Nov
Dec
Jan
Feb
Mar
Apr
Rooftop calibration
Fixed angle -3.5°
Hill-Ekström calibration
probGLS
BG074
BG902
BG905
BG913

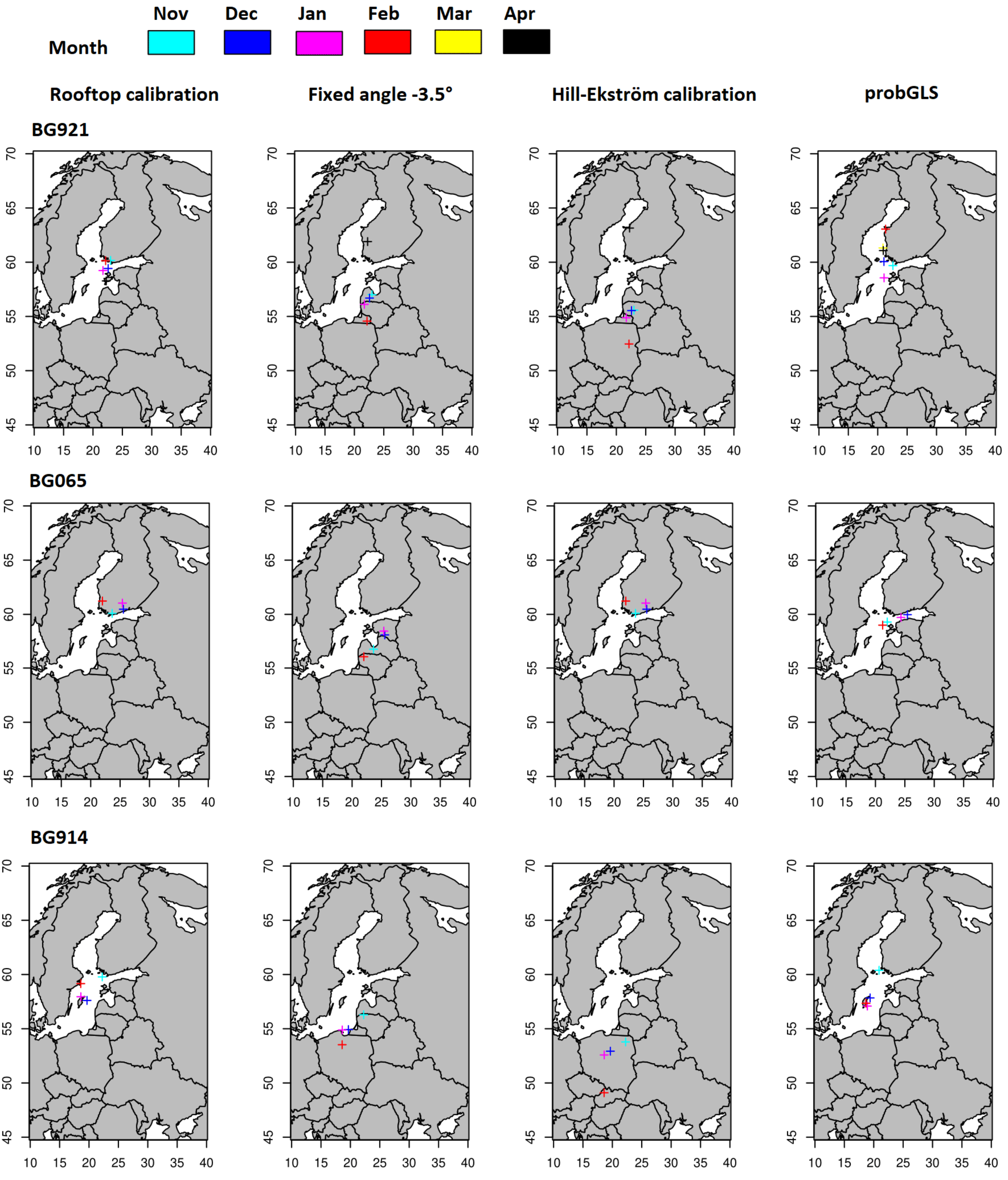

Month
Nov
Dec
Jan
Feb
Mar
Apr
Rooftop calibration
Fixed angle -3.5°
Hill-Ekström calibration
probGLS
BG921
BG065
BG914
